# Platelet glycoprotein IIb/IIIa inhibitor tirofiban in clopidogrel-naïve patients undergoing elective percutaneous coronary intervention

**DOI:** 10.1007/s12471-023-01810-2

**Published:** 2023-09-15

**Authors:** Zarina Habibi, Jasper Luijkx, Ben C. G. Gho, Mustafa Ilhan, Leo F. Veenstra, Lex A. W. Ruiters, Mera Stein, Arnoud W. J. van ’t Hof, Saman Rasoul

**Affiliations:** 1 Department of Cardiology, Zuyderland Medical Centre, Heerlen, The Netherlands; 2 Department of Cardiology, Maastricht University Medical Centre, Maastricht, The Netherlands

**Keywords:** Tirofiban, Clopidogrel, Elective percutaneous coronary intervention, Stable angina pectoris

## Abstract

**Background:**

The safety of administration of tirofiban, a platelet glycoprotein IIb/IIIa inhibitor, followed by a clopidogrel loading dose in clopidogrel-naïve patients undergoing ad-hoc percutaneous coronary intervention (PCI) is not yet clear.

**Methods:**

In a retrospective observational cohort analysis, clopidogrel-naïve patients undergoing ad-hoc PCI who received a high-dose bolus of tirofiban (25 μg/kg) followed by a 600-mg clopidogrel loading dose (group 1) were compared with patients undergoing elective PCI who were pretreated with clopidogrel (group 2), between September 2014 and October 2021. The primary outcome was major adverse cardiovascular events (MACE) defined as the composite of death, myocardial infarction, stroke, target-lesion revascularisation and bleeding at 30 days. Secondary outcomes were MACE at 7 days and individual components of the primary outcome at 7 and 30 days.

**Results:**

A total of 1404 patients were included: 432 (31%) in group 1 and 972 (69%) in group 2. Median age was 69 years, and 28% were female. At 7‑day follow-up, MACE occurred in 1.4% in group 1 versus 3.0% in group 2 (*p* = 0.08). 30-day MACE were observed in 1.9% in group 1 and 4.2% in group 2 (*p* = 0.03). Secondary outcomes were comparable between the groups. Cox regression analysis, corrected for baseline differences, revealed no significant difference in the primary outcome (hazard ratio: 1.8; 95% confidence interval: 0.8–3.9).

**Conclusion:**

Ad-hoc PCI in clopidogrel-naïve patients who were treated with high-dose bolus of tirofiban followed by a clopidogrel loading dose immediately after the procedure appeared to be safe.

## What’s new?

- A high-dose bolus of tirofiban (25 μg/kg) followed by a 600-mg loading dose of clopidogrel in clopidogrel-naïve patients undergoing ad-hoc percutaneous coronary intervention (PCI) did not have a significant effect on the incidence of major adverse cardiovascular events compared with patients undergoing elective PCI who were pretreated with clopidogrel ≥ day prior.
- Performing ad-hoc PCI in clopidogrel-naïve patients undergoing elective PCI who were treated with a single high-dose bolus of tirofiban followed by a clopidogrel loading dose directly after the procedure was safe.
- Further study is necessary to determine the optimal antithrombotic therapy for ad-hoc PCI in clopidogrel-naïve patients.

## Introduction

In patients with angina pectoris (AP), optimal medical therapy is strongly recommended to reduce symptoms, slow the progression of atherosclerosis and prevent atherothrombotic events [1]. Invasive coronary angiography (ICA) is recommended in patients with a high clinical likelihood of coronary artery disease, severe symptoms refractory to medical therapy or typical AP at a low level of exercise and a clinical evaluation that indicates high event risk [1].

To relief symptoms and/or improve the prognosis, revascularisation plays a crucial role in the management of AP, on top of medical treatment. In case of revascularisation by percutaneous coronary intervention (PCI), platelet inhibitors should be started prior to the procedure [2]. The strategy of platelet aggregation inhibition may vary. The European Society of Cardiology recommends aspirin and clopidogrel pretreatment for elective PCI procedures and reserves the use of platelet glycoprotein IIb/IIIa inhibitors (GPIs) such as tirofiban only for specific ‘bail-out’ situations [2]. In contrast, the 2011 ACCF/AHA/SCAI Guideline for Percutaneous Coronary Intervention states that in patients undergoing elective PCI who are treated with unfractionated heparin (UFH) and not pretreated with clopidogrel, administration of a GPI is reasonable and might be reasonable in those who are adequately pretreated [3].

Clopidogrel has a duration of action of ≥ 6 h. The literature is ambiguous as to when to administer clopidogrel in the setting of ICA, as the anatomy of the coronaries and thus the need for revascularisation are not always known in patients undergo ICA. Furthermore, administration of clopidogrel > 6 h prior to ICA may lead to bleeding complications [4]. However, Sabatine et al. found clopidogrel pretreatment before PCI to be beneficial, and this was not associated with a significant excess of thrombolysis in myocardial infarction major or minor bleeding [5].

**Fig. 1:**
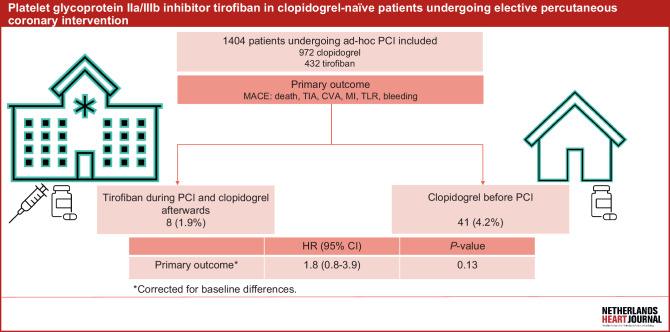
Platelet glycoprotein IIa/IIIb inhibitor tirofiban in clopidogrel-naïve patients undergoing elective percutaneous coronary intervention. (*PCI* percutaneous coronary intervention, *MACE* major cardiovascular events, *TIA* transient ischaemic attack, *CVA* cerebrovascular accident, *MI* myocardial infarction, *TLR* target-lesion revascularisation, *HR* hazard ratio)

To bridge the duration action of clopidogrel when it is administered immediately after PCI in clopidogrel-naïve patients, a high-dose bolus (25 μg/kg) tirofiban may be administered intravenously prior to the PCI. In selected patients with acute coronary syndrome (ACS) undergoing PCI, GPIs reduce the number of ischaemic events occurring during or after the intervention [6]. At present, it is not yet clear whether a high-dose bolus of a GPI in clopidogrel-naïve patients with stable AP undergoing ad-hoc PCI is as safe and effective. We aimed to evaluate the safety and effectivity of a high-dose bolus of tirofiban followed by a 600-mg loading dose of clopidogrel in clopidogrel-naïve patients undergoing ad-hoc (unplanned) PCI (Fig. 1).

## Methods

### Study design and participants

This was a retrospective cohort study with participants from Zuyderland Medical Centre in Heerlen, the Netherlands. The study population consisted of patients undergoing elective or ad-hoc PCI from 22 September 2014 to 1 October 2021. Eligible patients were ≥ 18 years of age who had AP or angina-equivalent symptoms and were admitted to the Cardiology Outpatient Department. Patients with ACS in the previous year and those with chronic total occlusion were excluded. All patients undergoing PCI at the Zuyderland Medical Centre are prospectively registered in a database.

The investigation conformed to the principles outlined in the Declaration of Helsinki. The study was approved by the Committee on Research Ethics of the Zuyderland Medical Centre.

### Intervention

The study population was divided into 2 groups. Group 1 consisted of clopidogrel-naïve patients underwent ad-hoc PCI who received high-dose bolus of tirofiban intravenously in the cardiac catheterisation room, i.e. immediately after ICA but before the start of the PCI procedure, followed by a 600-mg loading dose of clopidogrel immediately after the intervention (group 1). Group 2 comprised patients who received clopidogrel 75 mg daily in the outpatient setting ≥ 1 day before elective PCI (group 2).

### Outcome assessment

The primary outcome comprised major adverse cardiovascular events (MACE) at 30 days after adjustment for baseline differences. MACE was defined as the composite of death, myocardial infarction (MI), transient ischaemic attack (TIA), cerebrovascular accident (CVA), target-lesion revascularisation and bleeding. MI was defined as a significant troponin rise and/or fall in the clinical blood assessment [7]. Bleeding was defined according to the definition of the Bleeding Academic Research Consortium (BARC) criteria from type 3 onwards. Type 3a is defined as bleeding resulting in a haemoglobin drop of 3 to < 5 g/dl, type 3b as bleeding plus a haemoglobin drop ≥ 5 g/dl and type 3c as intracranial haemorrhage [8].

Secondary outcomes consisted of MACE at 7 days and the individual components of the primary outcome at 7 days and after 30 days. All outcomes were evaluated using electronic patient records.

### Statistical analysis

Baseline characteristics were analysed for normal distribution using the Skewness and Kurtosis test. Differences in baseline characteristics between the 2 groups were compared using the chi-squared test for categorical variables and the Mann-Whitney U test for continuous variables. Data are presented as mean ± standard deviation for continuous variables and as median (interquartile range) or frequency and percentage for categorical variables.

Based on the PRAGUE-8 trial, we concluded that a combined endpoint of death, MI, cerebral infarction and re-intervention within 1 week is 1.3% in patients who received a loading dose of clopidogrel ≥ 6 h before CAG and PCI. Few data are available on the combination of ad-hoc bolus GPI in the elective setting with subsequent clopidogrel loading. Marian et al. conducted an RCT comparing a bolus GPI with powdered ticagrelor in patients with IAP. None of the patients who received ad-hoc bolus GPI and a 600-mg clopidogrel loading dose after PCI, had the primary outcome measure [4, 10]. Based on the studies mentioned, it could be expected that in the clopidogrel group in our study approximately 1.3% of the patients will reach the primary endpoint after 1 week. For the ad-hoc GPI group, this would be between 99 and 99.9%. With a two-sided significance level of 2.5%, a power of 80% and a non-inferiority limit of 1%, a sample size of 432 patients per treatment group and 864 patients in total was calculated.

To compare outcome measures, the chi-squared test was performed. Results with a *p*-value < 0.05 were considered statistically significant. Cox hazard regression analysis was performed to calculate the hazard ratio (HR) with 95% confidence interval (CI) for the primary outcome corrected for significant baseline characteristics. Results for the primary outcome were analysed with the log-rank test and are presented as Kaplan-Meier survival curves. All statistical analyses were performed using the IBM SPSS Statistics 25 programme for Windows (SPSS Inc, Armonk, NY, USA).

## Results

### Baseline characteristics

During the study period, 1404 patients met the in- and exclusion criteria and were included in this analysis: 432 (31%) in group 1 and 972 (69%) in group 2. Their baseline characteristics are presented in Tab. 1. The mean age was 69 ± 9 years, 396 patients (28%) were female, and 406 (29%) had diabetes mellitus. Significant differences between the groups were observed for age, number of patients with hypercholesterolaemia, medical history (coronary artery bypass grafting, TIA, CVA), estimated glomerular filtration rate, creatinine level and PCI access site.

**Table 1:** Baseline characteristics of study population

Characteristic	Group 1 (*n* = 432)^a^	Group 2 (*n* = 972)^b^	*P*-value^c^
Age, years	66 ± 9	69 ± 10	< 0.001
Male	304 (70)	704 (72)	0.429
BMI, kg/m^2^	28 (25–31)	28 (25–31)	0.846
**Medical history**			
Hypertension	325 (75)	774 (80)	0.065
Hypercholesterolaemia	348 (81)	827 (85)	0.034
Smoking	82 (19)	167 (17)	0.556
Diabetes mellitus	116 (27)	290 (30)	0.255
MI > 1 year prior	131 (30)	323 (33)	0.283
PCI > 1 year prior	138 (32)	310 (32)	0.985
CABG	47 (11)	166 (17)	0.003
TIA	18 (4)	82 (8)	0.004
CVA	9 (2)	69 (7)	< 0.001
*History of bleeding*			0.326
– Gastrointestinal bleeding	7 (2)	15 (2)	
– Haemorrhagic stroke	0 (0)	5 (0.5)	
**Procedure**			
*Access site*			0.010
– Femoral	36 (8)	135 (14)	
– Radial	396 (92)	836 (86)	
– Ulnar	0	1 (0.2)	
Tirofiban bail-out	16 (4)	32 (3)	0.794
Use of acetylsalicylic acid	423 (98)	937 (96)	0.155
Use of oral anticoagulants	57 (13)	155 (16)	0.551
*Laboratory*			
Creatinine, µmol/l	86 (74–101)	90 (78–106)	< 0.001
eGFR, ml/min per 1.73 m^2^	68 (60–86)	60 (56–77)	< 0.001
Haemoglobin, mmol/l	8.9 (8.1–9.5)	8.8 (8.1–9.4)	0.077
LVEF, %	55 (45–60)	55 (45–60)	0.731

Data are mean ± standard deviation, median (interquartile range) or *n* (%)

*MI* myocardial infarction, *CABG* coronary artery bypass grafting, *TIA* transient ischaemic attack, *CVA* cerebrovascular accident, *eGFR* estimated glomerular filtration rate, *LVEF* left ventricular ejection fraction

^a^Group 1: clopidogrel-naïve patients who received high-bolus dose of tirofiban (25 µg/kg) before undergoing ad-hoc percutaneous coronary intervention (*PCI*), followed by 600-mg loading dose of clopidogrel

^b^Group 2: patients undergoing elective PCI who were pretreated with clopidogrel

^c^Group differences were tested with Mann-Whitney U test or chi-squared test

### Outcomes

At 7 days of follow-up, the primary composite outcome (MACE) had occurred in 6 patients (1.4%) in group 1 and 30 patients (3.0%) in group 2 (*p* = 0.08). At 30-day follow-up, MACE were observed in 8 patients (1.9%) in group 1 compared with 41 (4.2%) in group 2 (*p* = 0.03) (Tab. 2). Acute stent thrombosis occurred in 3 patients (0.3%) in group 2 and none in group 1. After adjusting for baseline differences, there was no significant difference in the primary outcome (HR: 1.8; 95% CI: 0.8–3.9) (Tab. 3).

**Table 2:** Outcomes at 7 and 30 days

Outcome	Group 1 (*n* = 432)^a^	Group 2 (*n* = 972)^b^	*P*-value^c^
*7 days*			
– MACE^d^	6 (1.4)	30 (3.0)	0.08
– Death	1 (0.2)	5 (0.5)	0.45
– TIA	0 (0)	2 (0.2)	0.35
– CVA	0 (0)	3 (0.3)	0.25
– MI	3 (0.7)	15 (1.5)	0.19
– TLR	2 (0.5)	1 (0.2)	0.18
– Bleeding (BARC ≥ 3)	1 (0.2)	4 (0.4)	0.60
*30 days*			
– MACE^d^	8 (1.9)	41 (4.2)	0.03
– Death	1 (0.2)	7 (0.7)	0.26
– TIA	0 (0)	2 (0.2)	0.35
– CVA	0 (0)	3 (0.3)	0.25
– MI	5 (1.2)	22 (2.3)	0.16
– TLR	2 (0.5)	2 (0.2)	0.40
– Bleeding (BARC ≥ 3)	1 (0.2)	8 (0.8)	0.20

Data are *n* (%)

^a^Group 1: clopidogrel-naïve patients who received high-bolus dose of tirofiban (25 µg/kg) before undergoing ad-hoc percutaneous coronary intervention (*PCI*), followed by 600-mg loading dose of clopidogrel

^b^Group 2: patients undergoing elective PCI who were pretreated with clopidogrel

^c^Group differences were tested with chi-squared test

^d^Major adverse cardiovascular events (*MACE*) were defined as death, transient ischaemic attack (*TIA*), cerebrovascular accident (*CVA*), myocardial infarction (*MI*), target-lesion revascularisation (TLR) and bleeding (according to Bleeding Academic Research Consortium (*BARC*) ≥ 3)

**Table 3:** Hazard ratios for primary outcome for group 1 versus group 2^a,b^

Variable	HR (95% CI)^c^	*P*-value
*Overall*	1.8 (0.8–3.9)	0.13
– Age	1.0 (1.0*–*1.1)	0.14
– Hypercholesterolaemia	1.0 (0.5–2.2)	0.98
– Prior CVA	0.6 (0.2–1.5)	0.27
– Prior CABG	1.5 (0.6–3.6)	0.33
– eGFR	1.0 (1.0–1.0)	0.02

^a^Primary composite outcome comprised death, transient ischaemic attack, cerebrovascular accident (*CVA*), myocardial infarction, target-lesion revascularisation and bleeding (according to Bleeding Academic Research Consortium ≥ 3) at 30 days

^b^Group 1: clopidogrel-naïve patients who received high-bolus dose of tirofiban (25 µg/kg) before undergoing ad-hoc percutaneous coronary intervention (*PCI*), followed by 600-mg loading dose of clopidogrel. Group 2: patients undergoing elective PCI who were pretreated with clopidogrel

^c^Cox-hazard regression analysis was used to calculate hazard ratio (HR) with 95% confidence interval (CI) adjusted for baseline characteristics age, hypercholesterolaemia, medical history (coronary artery bypass grafting (*CABG*), CVA) and estimated glomerular filtration rate (*eGFR*)

**Fig. 2:**
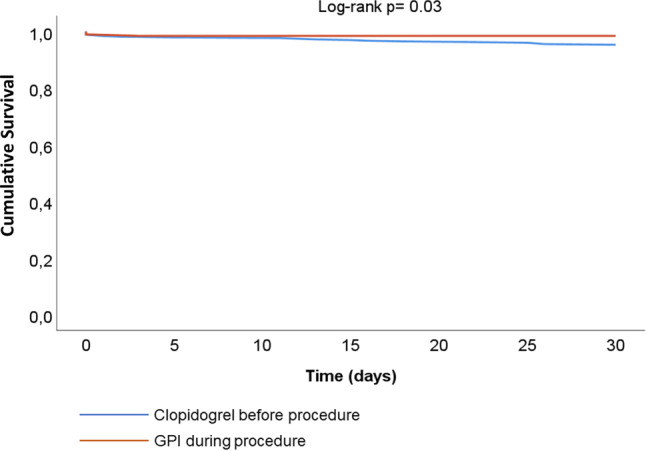
Kaplan-Meier curves for clopidogrel-naïve patients undergoing ad-hoc percutaneous coronary intervention (*PCI*) who received high-dose bolus of platelet glycoprotein IIb/IIIa inhibitor (*GPI*) tirofiban followed by a 600-mg clopidogrel loading dose and patients undergoing elective PCI who were pretreated with clopidogrel

Secondary outcomes were comparable between the groups (Tab. 2). Kaplan-Meier survival curves are shown in Fig. 2.

## Discussion

In this retrospective study, we evaluated the safety of ad-hoc PCI in clopidogrel-naïve patients who were treated with intravenous tirofiban followed by a 600-mg clopidogrel loading dose immediately after the PCI procedure. After adjusting for baseline differences, we found no significant differences in 30-day MACE between the use of clopidogrel ≥ 1 day prior to elective PCI compared with the use of tirofiban followed by a clopidogrel loading dose in clopidogrel-naïve patients undergoing ad-hoc PCI. In addition, there were so significant differences between the 2 groups in the secondary outcomes. These findings suggest that in clopidogrel-naïve patients, ad-hoc PCI shortly after administration of a single high-dose bolus of tirofiban followed by a 600-mg clopidogrel loading dose immediately after the PCI is safe and effective.

Over the past 2 decades, there has been a rise in the use of ad-hoc PCI, which can mainly be attributed to the efficacy of PCI for ACS and research indicating that ad-hoc PCI is a safe and effective option [9]. Possible advantages of ad-hoc PCI in clopidogrel-naïve patients with AP are reduction of the risk of access site-related complications, Furthermore, this strategy has proven to be cost-effective as it reduces the demand for materials, personnel and hospital capacity. Moreover, it is associated with greater patient-friendliness, as the patient is diagnosed and treated in one session [9].

Pretreatment with clopidogrel in all patients who undergo elective ICA may be safe. The PRAGUE-8 trial showed that a high loading dose of clopidogrel before elective ICA/PCI increased the risk of minor bleeding complications, while the benefit on periprocedural infarction was not significant [4]. The authors concluded that clopidogrel can be administered safely in the catheterisation laboratory. Furthermore, in patients undergoing elective or primary PCI, clopidogrel pretreatment is safe and effective [5].

Limited and ambiguous literature is available on the combination of a GPI bolus with subsequent P2Y_12_ inhibitor loading in the elective setting. The previously mentioned American and European guidelines do not agree on the use of a GPI in ad-hoc PCI [2, 3]. The European guidelines recommend using aspirin and clopidogrel as pretreatment for elective stenting procedures and UFH during PCI, whereas the American guideline states that in patients undergoing elective PCI who are treated with UFH and not pretreated with clopidogrel, it is reasonable to administer a GPI [2, 3]. Our results are in line with the recommendations of the American guidelines.

GPIs appear to reduce ischaemic events occurring after PCI in patients with ACS. The ischaemic benefit of GPI therapy has been attributed to its rapid onset of action and the > 80% platelet aggregation inhibition it induces in most patients [6]. Previous studies have shown the effectivity of GPIs in patients undergoing PCI [6]. In a post-hoc analysis, the relative efficacy and safety of ticagrelor versus clopidogrel in patients who did or did not receive a GPI in the PLATO trial were studied [5]. The authors found that the efficacy and safety of ticagrelor as compared with clopidogrel were not modified by GPI use according to the primary efficacy and safety endpoints. Other studies in the acute setting showed no significant effect of GPI use on outcomes with different anti-platelet strategies, including clopidogrel versus placebo [5].

Marian et al. studied the efficacy of crushed ticagrelor versus an eptifibatide bolus plus clopidogrel in 100 P2Y_12_ inhibitor-naïve, troponin-negative patients with ACS and found that the eptifibatide bolus plus clopidogrel led to faster and more potent platelet inhibition than ticagrelor and reduced periprocedural MI and injury [10].

### Study limitations

Since this was a retrospective study, missing data could not be traced. Nonetheless, the study had enough power according to the calculated sample size. Furthermore, not all baseline characteristics were well balanced. Patients in group 2 were older, had a higher risk profile and had undergone more transfemoral procedures. Transfemoral access for performing coronary angiography may have a higher risk for bleeding complications [11]. In our study, we evaluated bleeding BARC ≥ 3; however, assessment of bleeding BARC 2 may at times also be important. Another limitation of our study is that we did not investigate the angiographic characteristics of the lesions.

Nevertheless, to reduce confounding bias, we performed Cox regression analysis. All patients who underwent elective invasive catheterisation were screened. The study sample can be considered representative of the population of patients undergoing PCI in daily practice. Performing ad-hoc PCI was at the discretion of the operator, while elective PCI was previously scheduled and usually discussed by the Heart Team. This may also have affected our findings. However, Cox regression analysis was performed to correct for important differences.

In the current study, clopidogrel-naïve patients were treated with a GPI bolus to bridge the duration action of clopidogrel. However, further research is needed to investigate the optimal pretreatment option for clopidogrel-naïve patients undergoing ad-hoc PCI, especially to analyse the safety of ad-hoc PCI in clopidogrel-naïve patients without GPI pretreatment or administration of a clopidogrel loading dose in the catheterisation laboratory immediately before or after the PCI.

### Clinical implications

This study may give an indication of the safety of GPI usage in the ad-hoc PCI setting in clopidogrel-naïve patients. Further study is necessary to determine the optimal antithrombotic therapy for ad-hoc PCI in clopidogrel-naïve patients.

## Conclusion

Performing ad-hoc PCI in clopidogrel-naïve patients who were treated with a single high-dose bolus of tirofiban followed by a loading dose of clopidogrel immediately after the procedure was safe.
